# Isotope niche dimension and trophic overlap between bigheaded carps and native filter-feeding fish in the lower Missouri River, USA

**DOI:** 10.1371/journal.pone.0197584

**Published:** 2018-05-21

**Authors:** Jianzhu Wang, Duane Chapman, Jun Xu, Yang Wang, Binhe Gu

**Affiliations:** 1 Engineering Research Center of Eco-environment in Three Gorges Reservoir Region, Ministry of Education, China Three Gorges University, Yichang, China; 2 U.S. Geological Survey, Columbia Environmental Research Center, Columbia, MO, United States of America; 3 Institute of Hydrobiology, the Chinese Academy of Sciences, Wuhan, China; 4 Department of Geological Sciences, Florida State University & National High Magnetic Field Laboratory, Tallahassee, FL, United States of America; 5 Soil and Water Science Department, University of Florida, Gainesville, Florida, United States of America; Texas A&M University, UNITED STATES

## Abstract

Stable carbon and nitrogen isotope values (δ^13^C and δ^15^N) were used to evaluate trophic niche overlap between two filter-feeding fishes (known together as bigheaded carp) native to China, silver carp (*Hypophthalmichthys molitrix*) and bighead carp (*Hypophthalmichthys nobilis*), and three native filter-feeding fish including bigmouth buffalo (*Ictiobus cyprinellus*), gizzard shad (*Dorosoma cepedianum*) and paddlefish (*Polyodon spathula*) in the lower Missouri River, USA, using the Bayesian Stable Isotope in R statistics. Results indicate that except for bigmouth buffalo, all species displayed similar trophic niche size and trophic diversity. Bigmouth buffalo occupied a small trophic niche and had the greatest trophic overlap with silver carp (93.6%) and bighead carp (94.1%) followed by gizzard shad (91.0%). Paddlefish had a trophic niche which relied on some resources different from those used by other species, and therefore had the lowest trophic overlap with bigheaded carp and other two native fish. The trophic overlap by bigheaded carp onto native fish was typically stronger than the reverse effects from native fish. Average niche overlap between silver carp and native species was as high as 71%, greater than niche overlap between bighead carp and native fish (64%). Our findings indicate that bigheaded carps are a potential threat to a diverse and stable native fish community.

## Introduction

Trophic structure of a biological community was typically described in the past as the number of species, size range, abundance and feeding habits. However, this information seldom provides significant insights as to the stability and function of a consumer community for biological conservation and management. The effects of biological invasion on a consumer community must be quantified not only from the viewpoint of species abundance and richness, but also from the interactions of introduced species with the native community. New theory and methodologies have helped increase understanding of ecosystem structure and function under various scenarios of natural conditions and anthropogenic disturbances [1–3].

Stable isotope natural abundance provides insights into carbon sources and trophic positions of consumers in aquatic food webs. Stable carbon isotopes (δ^13^C) of organic matter of different origins are often distinct from one another due to differences in photosynthetic pathways and growth conditions [4]. Transfers of organic matter to consumers do not result in substantial isotope fractionation and hence δ^13^C is used as an indicator for carbon flow and resource diversity. Stable nitrogen isotopes (δ^15^N) are useful in determining consumer trophic position because δ^15^N increases during each trophic transfer [3, 5]. Furthermore, consumer δ^13^C and δ^15^N provide a record of animal feeding and incorporation of dietary carbon and nitrogen during growth, thereby providing more accurate and integrated information on animal use of resources and trophic connections at different time and space scales.

Early use of stable isotope data in trophic ecology was limited to tracking carbon flow and to describing feeding relationships [6–8]. Later applications of stable isotope trophic ecology include the quantifications of food chain length [9, 10], dietary overlaps [11] and animal invasion [10]. With the introduction of advanced statistical tools, consumer stable isotopes are used to quantify omnivory, trophic niche, and niche overlap [12–14]. Layman [15] proposed a set of trophic niche metrics to describe community trophic structure. A number of studies have used this approach to quantify the changes in food web structure [16] as the results of ecosystem fragmentation [17], hydrological changes [18] and trophic niche overlap between native and nonnative fish [19]. More recently, a Bayesian approach has been used to quantify trophic metrics proposed by Layman [15] at species and community levels [20, 21]. This approach has also been used to identify patterns of trophic structure [22], species invasion [21, 23] and trophic overlap [24, 25].

The introduction of fish into aquatic ecosystems leads to changes in species composition, relative abundance and use of native resources. Risk assessments [12, 26, 27] have consistently predicted that bighead carp (*Hypopthalmithys nobilis*) and silver carp (*H*. *molitrix*) are highly likely to cause environmental problems where they invade and reach high abundance. Introduction of fishes of the genus *Hypophthalmichthys* (known together as bigheaded carps) around the world, including in North America, have consistently resulted in large reductions in the abundance of crustacean zooplankton and changes in the abundance of other plankton, including substantial changes in other aspects of the plankton community [27–29], commonly including changes in size of the phytoplankton community towards nano- and picophytoplankton, which are likely too small to be effectively preyed upon by North American filter-feeding fishes [30]. Indeed, the invasion and extreme population growth of silver carp (*Hypophthalmichthys molitrix*) and bighead carp (*Hypophthalmichthys nobilis*) in the Mississippi River basin has coincided with declines in condition [31, 32] and abundance [33, 34] of native planktivores including bigmouth buffalo (*Ictiobus cyprinellus*), gizzard shad (*Dorosoma cepedianum*) and emerald shiner (*Notropis atherinoides*). The recreationally important filter-feeder American paddlefish (*Polyodon spathula*) has been shown to be outcompeted by bighead carp in ponds [35]. Effects on other unstudied species are likely. Millions of dollars are spent annually in North America for the control of bighead and silver carp population and range. If diets overlap and resources are limiting, then competition between bigheaded carps and native fishes is likely.

**Fig 1:**
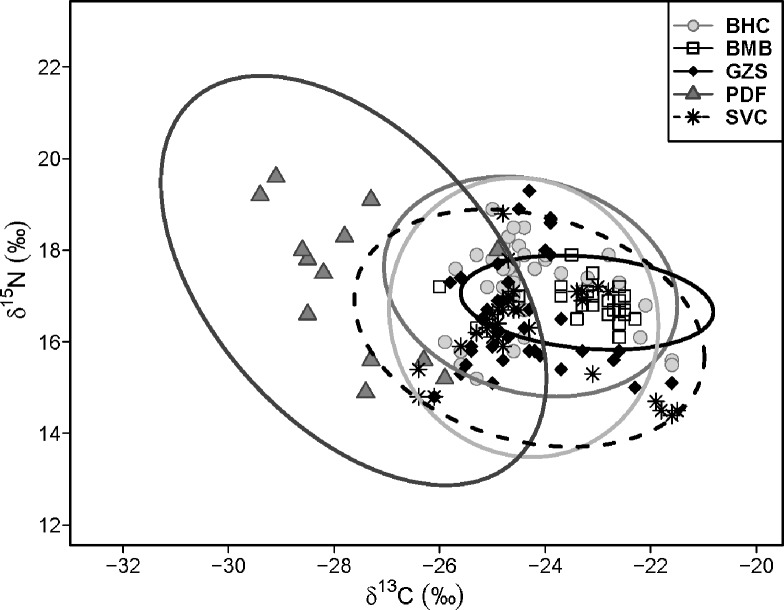
Sample collection site map for the lower Missouri River and adjacent tributaries, Missouri, USA.

The objective of this study was to elucidate niche characteristic and trophic overlaps of two bigheaded carps with three native fishes in the lower Missouri River (Fig 1). This was accomplished by calculating trophic niche metrics and overlap using the Bayesian stable isotope approach [20, 36]. Information discussed in this study might add important insights of potential effects of biological invasion on native species and management of natural ecosystems.

## Materials and methods

All fish handling and euthanization was performed according to relevant guidelines and regulations and the Columbia Environmental Research Center Animal Care and Use Policy. Experimental protocols were approved by the Columbia Environmental Research Center’s Animal Care and Use Committee and this study was approved by the committee.

### Sampling and laboratory analysis

Fish were collected in November and December of 2005. The majority of fish samples were collected from the Missouri River in central Missouri. A small number of fish were collected from within the Lamine and Osage Rivers, near their confluences with the Missouri River (Fig 1). There was a total of approximately 121 river km between the most distant sample sites, including distance up the tributaries. Fish were collected using trammel nets set in the low water-velocity environments preferred by all of these fish [37] and were measured to the nearest millimeter (total length, except paddlefish, where the eye-to-fork measurement was used). A sample of white muscle tissue was taken from above the lateral line and posterior to the dorsal fin of each fish, rinsed with deionized water, and placed in a scintillation vial. All samples were placed on ice for transport back to laboratory. To calculate fish trophic position, pink papershell (*Potamilus ohiensis*), a freshwater mussel, was collected as a baseline organism. The adductor muscle tissue was used, cleaned with deionized water and placed in a scintillation vial.

Muscle tissue was freeze-dried and powdered at the USGS Columbia Environmental Research Center at Columbia, MO. Approximately 1 mg of fine powder was loaded into a tin capsule for analysis. All samples were analyzed using a Carlo Erba Elemental Analyzer interfaced to a Finnigan MAT Delta Plus XP stable isotope ratio mass spectrometer at the Florida State University. The results were reported in the standard δ notation relative to the Vienna Pee Dee Belemnite (PDB) standard for ^13^C/^12^C and atmospheric nitrogen for ^15^N/^14^N ratios, respectively. The analytical precision (based on replicate analyses of lab standards processed with each batch of samples and on sample replicates) was ± 0.1‰ for both ^13^C and ^15^N analysis.

### Data analysis

Stable isotope data from the five filter-feeding fish were used to characterize trophic structure and potential resource competition among filter-feeding fish in the Lower Missouri River. Among them bighead and silver carp are considered invasive and the other three (gizzard shad, bigmouth buffalo and paddlefish) are native to the Lower Missouri River (Table 1). We only obtained sufficient samples of silver carp and gizzard shad to explore the difference and similarity of δ^13^C and δ^15^N across all locations. Results indicate that the relative proportions of δ^13^C and of δ^15^N for silver carp and gizzard shad collected from 2 to 3 river sections do not show significant difference within each species. Therefore, all data from same species collected from different locations were pooled for trophic niche analysis.

**Table 1:** Means and standard deviation (SD) of total length (mm) and stable carbon and nitrogen isotopes (‰), number of samples for each species collected from the lower Missouri River.

Species	Code	Total length		δ^13^C		δ^15^N		N
		mean	SD	mean	SD	mean	SD	
Bighead carp	BHC	745	78	-25.8	1.3	16.5	0.9	30
Silver carp	SVC	771	53	-27.2	1.0	14.8	0.7	25
Bigmouth buffalo	BMB	563	111	-25.4	0.8	16.1	0.5	17
Gizzard shad	GZS	35	3	-25.3	2.0	14.1	1.6	20
Paddlefish	PDF	800^a^	114	-27.6	1.3	17.3	1.6	13

Trophic position of fish is estimated as TP = (δ^15^NF - δ^15^NB)/Δ δ^15^N+2, where δ^15^NF is the mean δ^15^N value of a fish species, δ^15^NB is the mean δ^15^N value of the baseline organism which is pink papershell in this analysis (S1 Table). Δ δ^15^N is the nitrogen stable isotope fractionation factor per trophic transfer during animal feeding. We used the mean value of 3.4‰ reported by Minagawa and Wada [5]. The trophic level of pink papershell is 2 (primary consumer).

All data were tested for normality (Shapiro-Wilk test) before further statistical analysis (α = 0.05 in all cases). Kruskal-Wallis One Way Analysis of Variance on Ranks was used to detect differences between species. Except for R Bayesian analysis, all other statistical analyses were performed in Sigma Plot 13. (Systat Software, Inc, San Jose, CA). Five population niche metrics derived from stable isotope data were used to evaluate trophic structure of the invasive and native filter-feeding fish during this study. These metrics were adapted from community wide metrics proposed by Layman [15] and were calculated using the Bayesian approach in R21. The six niche metrics are measures of the total extent of spacing and trophic redundancy within a δ^13^C and δ^15^N bi-plot for community or species. The δ^13^C range (CRB) is the difference between the individuals with the most enriched and most depleted δ^13^C values and is a measure of basal resource diversity. The δ^15^N range (NRB) is the difference between the species with the most enriched and most depleted δ^15^N values and is a measure of trophic length within a population. The total area (TA) of the δ^13^C and δ^15^N bi-plot space describes the total space occupied by a population and was calculated using the standard Ellipse Area (SEAB). The mean distance to the δ^13^C- δ^15^N centroid (CDB) provides a measure of trophic diversity. The mean nearest neighbor distance (MNNDB) is the mean of the Euclidean distances to each species’ nearest neighbor in bi-plot space and thus a measure of the overall density of species packing. Small MNNDB suggests increased trophic redundancy. Finally, the standard deviation of nearest neighbor distance (SDNNDB) is a measure of the evenness of species packing in bi-plot space. Low SDNNDB values mean more even distribution of trophic niches.

To measure the trophic niche size and to test whether trophic niche overlap were not equivalently weighted among species, we used a probabilistic method developed by Swanson *et al*. [36]. The method measures a given 95% (or user-defined α) probability niche size and provides directional estimates of pairwise niche overlap in multivariate space. Swanson *et al*. [36] defined the niche overlap of species A onto species B as the fraction of the intersection area between niche A and niche B over the total niche area of B and vice versa. We used a 95% probability niche size and overlap for results and discussion.

## Results

A total of 104 fish belonging to the five species were collected from the lower Missouri River between the confluence of the Lamine River, nine river km upstream of Boonville, Missouri and the confluence of the Osage River, 22 km downstream of Jefferson City, Missouri or within those two tributaries, near their confluences with the Missouri (S1 Table). Sample size and mean δ^13^C and δ^15^N values of each fish species can be found in Table 1. Silver carp, bighead and paddlefish shared similar size range followed by bigmouth buffalo and gizzard shad which had the smallest size as adults among the five filter feeders (Table 1). The δ^15^N values differed significantly among species (Kruskal-Wallis One-way ANOVA, H = 19.79, df = 4, p<0.001). The highest mean value was found in paddlefish and the lowest in gizzard shad (Table 1). The δ^13^C values also differed significantly among species (Kruskal-Wallis One-way ANOVA, H = 48.77, df = 4, p<0.05). The highest mean value was found in gizzard shad and the lowest in paddlefish (Table 1).

### Population metrics

δ^15^N in four of the five species spanned a range (NRB) greater than the magnitude of the average isotope fractionation (3.4‰) per trophic transfer [5], indicating that some individuals of each species differed by one full trophic level (Table 2). Trophic position was lowest for gizzard shad (2.6) and highest for paddlefish (3.5) with an average of 3.1 for all species studied (Table 2). The δ^13^C in each species spanned a range (CRB) from 3.7 to 4.9‰ which is over 10-fold greater than the isotope fractionation (0.4‰) during each trophic transfer [9]. The CRB among species appeared more uniform than trophic length, with smallest CRB in bigmouth buffalo and greatest in silver carp.

**Table 2:** Bayesian trophic niche metrics and trophic position (TP) of two bigheaded carps and three native filter-feeding fish in the lower Missouri River.

Species	NR_B_	CR_B_	SEA_B_	CD_B_	MNND_B_	SDNND_B_	TP
Bighead	3.7	4.3	10.7	1.2	0.3	0.2	3.4
Silver carp	4.4	4.9	12.3	1.3	0.3	0.3	2.8
Bigmouth buffalo	1.8	3.7	4.4	0.8	0.3	0.3	3.4
Gizzard shad	4.5	4.5	11.6	1.4	0.3	0.2	2.6
Paddlefish	4.7	4.5	12.6	1.8	0.8	0.6	3.5

**Fig 2:**
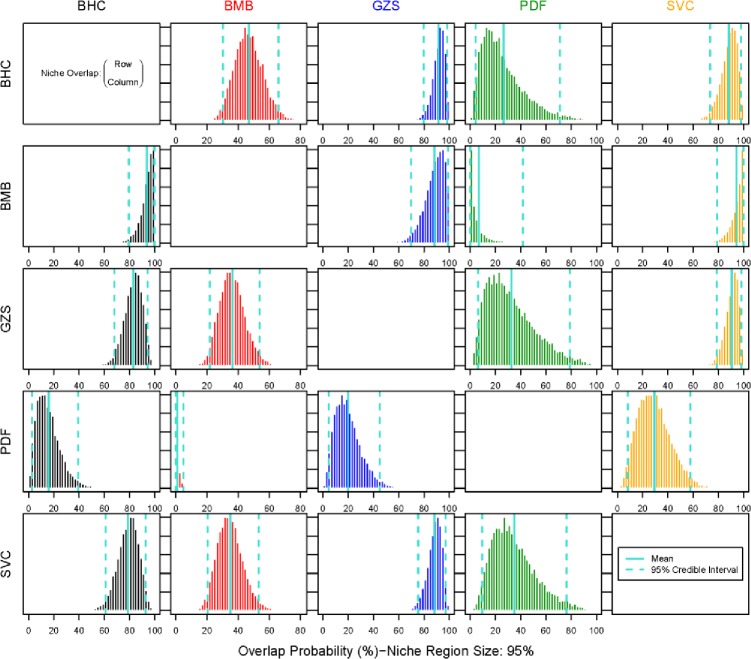
Dual stable isotope plot and 95% Bayesian standard ellipses (SEA_B_) of total trophic area for two invasive carps and three native fishes from the lower Missouri River. Please refer to Table 1 for species codes.

All species except for bigmouth buffalo shared similar niche space size (SEAB; Table 2 and Fig 2). Bigmouth buffalo had a much smaller niche space. As the result of small NRB and CRB, bigmouth buffalo had the lowest trophic diversity (CDB). Paddlefish possessed the greatest NRB and one of the highest CRB, and also displayed the highest CDB, higher MNNDB (low trophic redundancy) and SDNNDB (low trophic evenness) than other fish. CDB for other species other than bigmouth buffalo varied slightly. MNNDB and SDNNDB were nearly identical for all species except paddlefish (Table 2).

### Population niche overlap

**Fig 3:**
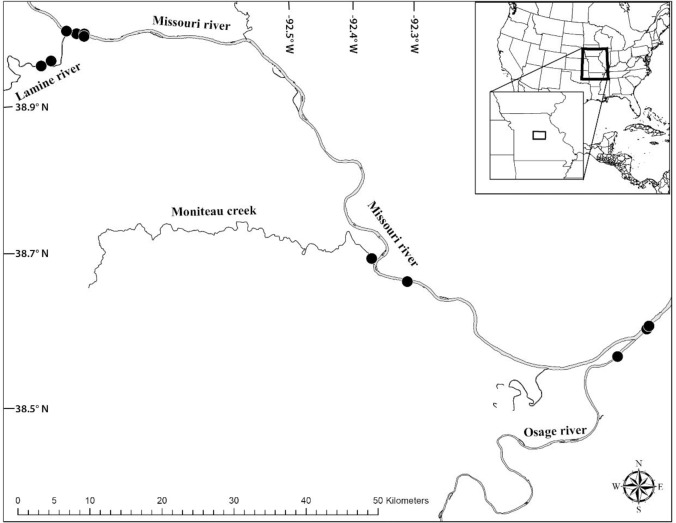
Probabilistic niche overlap (%) for a standard eclipse niche space (N_R_) of 95%. The means and 95% intervals are displayed in green. Please refer to Table 1 for species codes.

**Fig 4:**
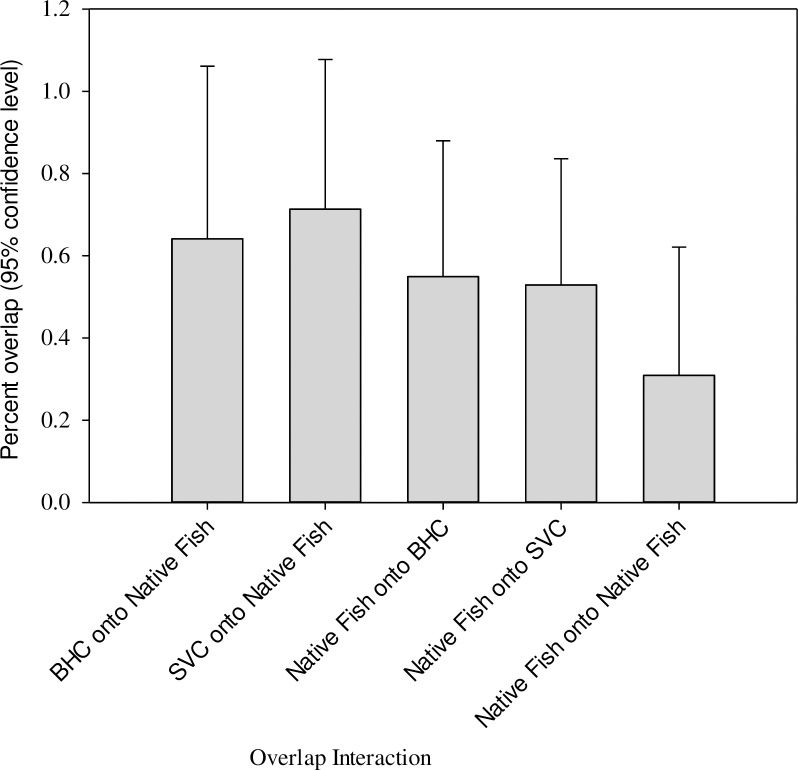
Average trophic overlap interactions of invasive and native fishes from the lower Missouri River. Please refer to Table 1 for species codes.

There are various degrees of trophic overlap between species and between invasive and native species with an overall average of 54% (Fig 3). High trophic overlaps (over 90%) are all involved with one of the invasive carp. The highest trophic overlap is found between silver carp and bigmouth buffalo (94.1%) and between bighead carp and bigmouth buffalo (93.6%) followed by gizzard shad onto bighead carp (91.3%) and silver carp onto gizzard shed (90.6%). The lowest trophic overlap between bigheaded carps and native fish is found between bighead and paddlefish (26.8%) while overlap between silver carp and paddlefish is higher (35.4%). Average trophic overlap between silver carp and native species was as high as 71%, greater than trophic overlap between bighead and native fish (64%) (Fig 4).

Trophic overlap between native fish averaged 31%, which is considerably lower than overlap between bigheaded carp and native fish, which averaged 67.7% (Fig 4) largely because of the different trophic space of paddlefish. The overlap between paddlefish and bigmouth buffalo, and between paddlefish and gizzard shad are 7.2% and 33.0%, respectively. However, trophic overlap between gizzard shad and bigmouth buffalo was as high as 88.0%. The Bayesian approach provided by Swanson *et al*. [36] allowed evaluation of species interactions for trophic overlap. The effects of trophic overlap by bigheaded carp on native fish were on average stronger than the effects of overlap by native fish (Fig 4). For example, trophic overlap of bighead carp onto bigmouth buffalo was 93.6% while the reverse was only 46.8%. High overlap in both directions was found between silver carp and gizzard (90.6 and 88.3% respectively). On the other hand, there were also cases in which native fish posed greater overlap onto bigheaded carps. The trophic overlap between bighead and gizzard shad was high but the effect of gizzard shad on bighead carp was slightly greater than the effect of bighead carp on gizzard shad. This also occurred in the interaction between bighead carp and paddlefish, although effects in both directions were minimal. However, the trophic overlap by silver carp onto native fish was all greater than the overlap from native fish (Fig 4).

## Discussion

This study revealed potentially high degree of trophic overlap between two bigheaded carps and three native filter-feeding fish in the Lower Missouri River. The probabilistic method for trophic overlap analysis developed by Swanson *et al*. [36] allows evaluation of species A onto species B or vice versa. Higher degrees of trophic overlap by both species of bigheaded carps with native fish (Fig 4) indicate that bigheaded carps were more competitive and aggressive in resource use than the native fish when resources are limiting. These findings provide additional insights of biological invasion ecology and management of invasive fish in US rivers.

The five species collected in this study have been reported to move long distances in US rivers [38–41], much longer than the distance between our sampling points, which may integrate the stable isotope signals of the natural resources used during their feeding movement. Our results are based on data collected in a single season during the study period. Since stable isotope signals in large fish reflect feeding and growth over a multiple year period, seasonal changes in dietary isotope signals have been incorporated into fish tissues and reveal the use of natural resources during their growth period. Annual collection of fish samples on a multiple year scale may reveal long-term changes in environmental conditions, use of natural resources, niche characteristics and trophic overlap between native and invasive fish [18, 24, 42].

Trophic metrics derived from dual stable isotope analysis may provide novel ways of quantifying interactions among populations in aquatic food webs [15,21]. Except for bigmouth buffalo, the trophic niche space, trophic diversity, individual trophic packing and redundancy for all species was similar. Similarly, Hill *et al*. [43] also found isotope metrics of the invasive fish were similar to or consistently mid-range in comparison with their native counterparts in the Nseleni River, South Africa. Resource competition depends largely on the intensity of trophic overlap between species and resource abundance. The resources available for water column filter feeders are limited to plankton and detritus in freshwaters. Bigheaded carps are well established in Missouri River [27] and are likely capable of fully utilizing the available natural resources. The similarity of trophic niche space between invasive and native fish in the Lower Missouri River would suggest that the two bigheaded carps were highly competitive, assuming a limited resource. Decrease in condition of gizzard shad in the Missouri River [31] since the invasion suggests that the planktonic food resource is now limiting. In addition, extremely rapid growth rates of bighead and silver carp in the Mississippi and Illinois Rivers were reported early in the invasion [44], but growth rates and condition factors have declined as the number of bigheaded carp has increased [45]. This is a likely indication of intraspecific or intrageneric competition, and thus of a limited resource in those nearby rivers, which are characterized by greater chlorophyll concentrations and higher planktonic productivity than the Missouri River [46]. However, without the knowledge of the trophic niche size of the native fish prior to the invasion of the carps, it is impossible to know if the current niche size has changed and is the result of resource competition with the bigheaded carps.

In other locations, silver carp has been found to depend on both phytoplankton and zooplankton while bighead carp is largely a zooplankton feeder [47–49]. Our estimates of trophic position for silver carp and bighead carp also confirmed their dietary preference in the lower Missouri River. The high trophic overlap between silver carp and bighead carp found in this study is supported by studies elsewhere [11, 47]. The SEAB occupied by both carps essentially encompassed the entire niche space occupied by bigmouth and gizzard shad. The greater SEAB in silver carp resulted from their greater use of both primary and secondary trophic level resources than bighead carp, and is reflected in its higher trophic overlap with native species than bighead carp.

The least trophic overlap was found between paddlefish and other filter-feeding fish including the two bigheaded carp. This finding is consistent with that reported by Sampson *et al*. [50] who found clear diet dissimilarity between the two bigheaded carp and paddlefish. Paddlefish displayed the lowest δ^13^C and highest δ^15^N among fish, indicating that they used a portion of resources with higher trophic position than those used by carp. Schrank *et al*. [35] reported negative effect on young paddlefish growth when held in ponds with young bighead carp. However, an examination of existing historic and more recent Missouri River paddlefish length/weight data from several sources did not identify a decrease in condition of paddlefish after the invasion of bighead and silver carp (Chapman, unpublished data). Gizzard shad did decrease in condition over a similar period [31]. Our data indicate that paddlefish can take advantage of Missouri River food resources with a higher trophic position than those consumed strongly by bighead carp and silver carp. If those higher trophic level foods were not available in the pond study by Schrank et al. [35], it would explain why the interaction in that study between bighead carp and paddlefish was detrimental to paddlefish. Sampson et al. [50] also found that paddlefish had a higher trophic position and less overlap with bighead carp or silver carp than the other native filter feeders in Illinois River backwaters. Paddlefish is listed as a species of special concern by US Fish and Wildlife Service [51], thus it is important to further study the trophic overlap between bighead carp, silver carp and Paddlefish under various growth stages and environmental conditions.

Gizzard shad is capable of feeding benthically and also filter-feeding on seston. Their diets include various plankton and large amount of detritus [52]. In this study, gizzard shad showed resource diversity and niche space similar to the invasive carp, but their trophic level is the lowest (2.5), which suggested that they are omnivores depending on both primary and secondary consumers in the Lower Missouri River. Therefore, shad displayed greater trophic overlap with silver carp than bighead carp, and this overlap is potentially responsible for the decline in gizzard shad condition [32] after the carp invasion.

Trophic overlap between paddlefish and other two native filter feeders was very small. This is not surprising because a community inhabiting in the same ecosystem must develop niche separation to avoid resource competition. This could be accomplished by using different resources and positioning at different trophic levels. For example, by using basal resources depleted in δ^13^C, paddlefish distanced themselves in SEAB from all other species. The niche space of bigmouth buffalo fell nearly completely within that of shad, while a large portion of gizzard shad’s niche space was located outside that of bigmouth buffalo, indicating high overlap by shad onto bigmouth buffalo but low overlap by bigmouth buffalo onto gizzard had. Bigmouth buffalo displayed smallest resource and trophic ranges, and trophic diversity than other species and occupied a narrow niche within the niche space occupied by both bigheaded carps in the lower Missouri River. Hence, their overlap was greatest among the filter feeding species community. Differences in isotopic niche among these fish appear to result from their differences in feeding habitats as discussed above.

Ecological effects of invasive fish, including silver carp and bighead carp, on native communities have also been reported elsewhere [53, 54]. However, because of the difficulties in providing replication or controls, and in working in such large and variable systems, little research has been performed that can validate the predictions [27] and perception [55] of undesirable effects on native species or link observed effects on native species [32] to the carp invasion. Our results show trophic overlap between the invasive and native filter feeding fishes especially for gizzard shad and bigmouth buffalo, and thus provide support for the hypothesis that bighead carp and silver carp have undesirable effects on native species in the Lower Missouri River. With the recent rapid increases in abundance of bighead and silver carp in the Mississippi River system [50], our data indicate a potential threat to biodiversity and to recreational and commercial fisheries in the major rivers of the United States.

## Supporting information

S1 Table Stable isotope data for fish and invertebrates from the lower Missouri River. (PDF)
