# Correction: Isotope niche dimension and trophic overlap between bigheaded carps and native filter-feeding fish in the lower Missouri River, USA

**DOI:** 10.1371/journal.pone.0199805

**Published:** 2018-06-22

**Authors:** Jianzhu Wang, Duane Chapman, Jun Xu, Yang Wang, Binhe Gu

The images for Figs 1, 2 and 3 are incorrectly ordered. The image that appears as Fig 1 should be Fig 2. The image that appears as Fig 2 should be Fig 3. The image that appears as Fig 3 should be Fig 1. The figure captions appear in the correct order.

**Fig 1:**
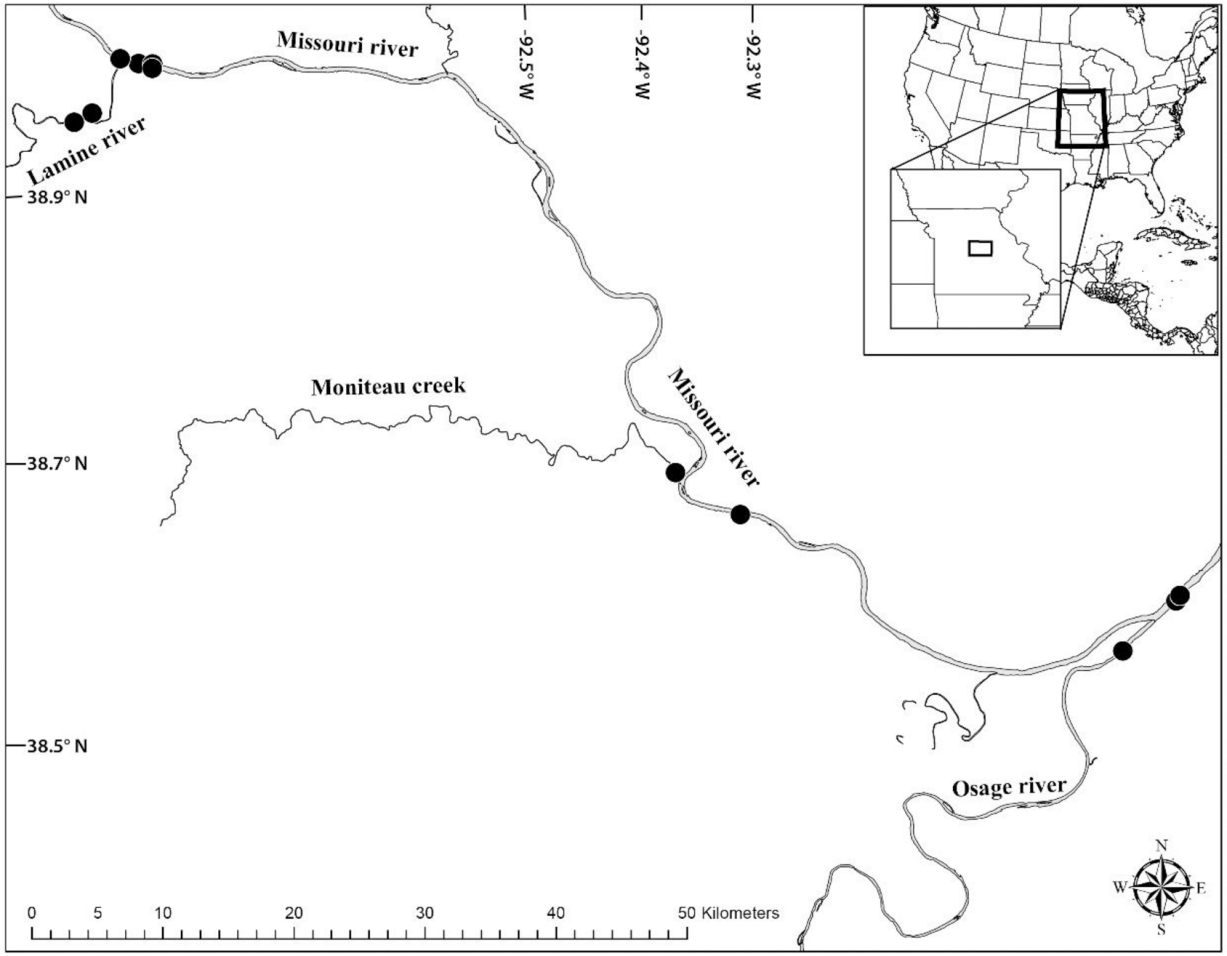
Sample collection site map for the lower Missouri River and adjacent tributaries, Missouri, USA.

**Fig 2:**
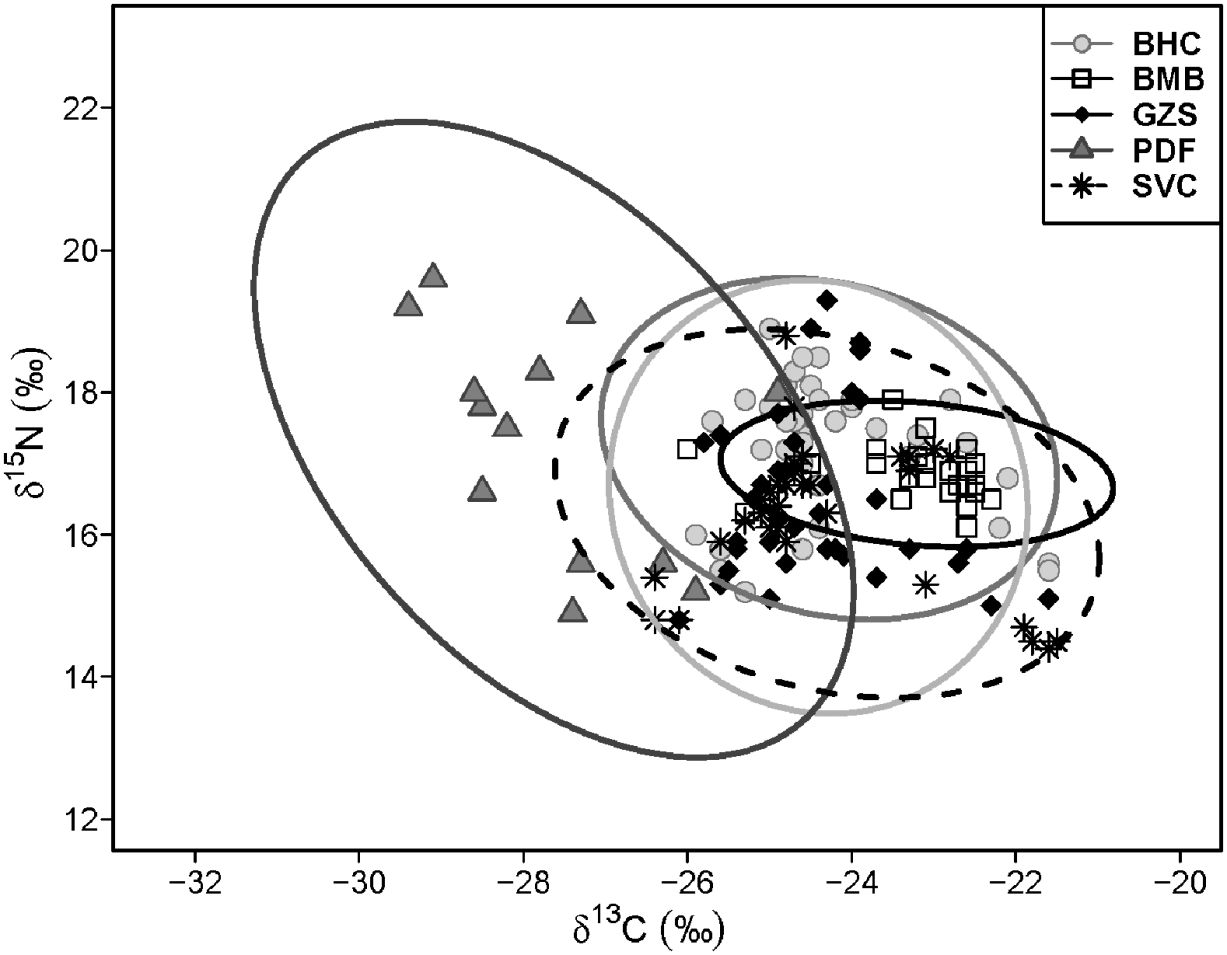
Dual stable isotope plot and 95% Bayesian standard ellipses (SEA_B_) of total trophic area for two invasive carps and three native fishes from the lower Missouri River. Please refer to Table 1 for species codes.

**Fig 3:**
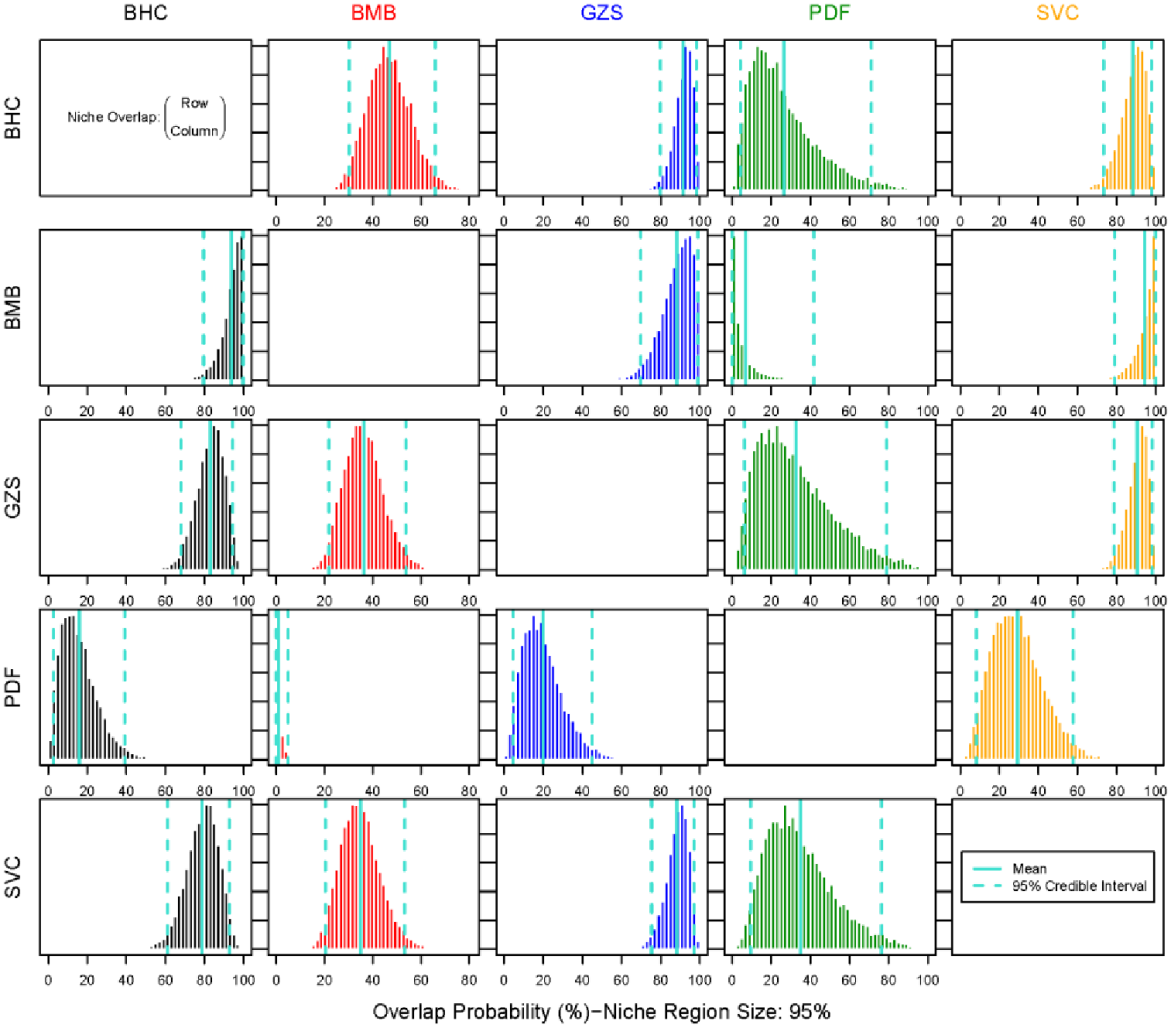
Probabilistic niche overlap (%) for a standard eclipse niche space (N_R_) of 95%. The means and 95% intervals are displayed in green. Please refer to Table 1 for species codes.
