# Supplementary material for: Isotope niche dimension and trophic overlap between bigheaded carps and native filter-feeding fish in the lower Missouri River, USA
Source: PLoS One. 2018 May 21;13(5):e0197584. doi: 10.1371/journal.pone.0197584 (PMC5962084; doi:10.1371/journal.pone.0197584)
Supplement: S1 Table — (PDF) [file pone.0197584.s001.pdf]

S1 Table (Wang et al.)

Species	Scientific name	Collection Date	Easting	Northing	UTM Zone	Length (mm)	$\delta^{15}N$ (‰)	$\delta^{13}C$ (‰)
Silver carp	*Hypophthalmichthys molitrix*	11/21/2005	514028	4314645	15 N	820	14.9	-28.0
Silver carp	*Hypophthalmichthys molitrix*	11/21/2005	514028	4314645	15 N	870	15.6	-28.5
Silver carp	*Hypophthalmichthys molitrix*	11/21/2005	514028	4314645	15 N	788	14.8	-27.5
Silver carp	*Hypophthalmichthys molitrix*	11/21/2005	514028	4314645	15 N	834	15.3	-28.0
Silver carp	*Hypophthalmichthys molitrix*	11/21/2005	514028	4314645	15 N	782	14.2	-28.6
Silver carp	*Hypophthalmichthys molitrix*	11/21/2005	514028	4314645	15 N	795	15.8	-27.2
Silver carp	*Hypophthalmichthys molitrix*	11/21/2005	514028	4314645	15 N	774	16.1	-28.5
Silver carp	*Hypophthalmichthys molitrix*	11/21/2005	514028	4314645	15 N	813	14.6	-27.1
Silver carp	*Hypophthalmichthys molitrix*	11/21/2005	514028	4314645	15 N	813	15.2	-26.4
Silver carp	*Hypophthalmichthys molitrix*	11/21/2005	514028	4314645	15 N	791	14.6	-27.8
Silver carp	*Hypophthalmichthys molitrix*	11/21/2005	514028	4314645	15 N	775	14.4	-26.4
Silver carp	*Hypophthalmichthys molitrix*	11/21/2005	514028	4314645	15 N	795	13.7	-26.0
Silver carp	*Hypophthalmichthys molitrix*	11/28/2005	510497	4310837	15 N	807	15.9	-28.6
Silver carp	*Hypophthalmichthys molitrix*	11/30/2005	515061	4314282	15 N	802	14.8	-26.8
Silver carp	*Hypophthalmichthys molitrix*	11/30/2005	515061	4314282	15 N	814	15.6	-29.0
Silver carp	*Hypophthalmichthys molitrix*	11/30/2005	515061	4314282	15 N	751	14.7	-25.2
Silver carp	*Hypophthalmichthys molitrix*	12/27/2005	589138	4269767	15 N	794	14.7	-27.7
Silver carp	*Hypophthalmichthys molitrix*	12/27/2005	589138	4269767	15 N	755	14.8	-27.1
Silver carp	*Hypophthalmichthys molitrix*	12/27/2005	589138	4269767	15 N	811	13.9	-25.8
Silver carp	*Hypophthalmichthys molitrix*	12/27/2005	589138	4269767	15 N	788	14.1	-26.8
Silver carp	*Hypophthalmichthys molitrix*	12/27/2005	589138	4269767	15 N	747	14.3	-27.5
Silver carp	*Hypophthalmichthys molitrix*	12/27/2005	589138	4269767	15 N	748	14.5	-26.5
Silver carp	*Hypophthalmichthys molitrix*	12/27/2005	589138	4269767	15 N	765	14.4	-26.3
Bigmouth buffalo	*Ictiobus cyprinellus*	11/21/2005	514028	4314645	15 N	330	16.6	-24.7
Bigmouth buffalo	*Ictiobus cyprinellus*	11/21/2005	510490	4310838	15 N	335	15.4	-25.4
Bigmouth buffalo	*Ictiobus cyprinellus*	11/21/2005	514028	4314645	15 N	621	16.7	-25.3
Bigmouth buffalo	*Ictiobus cyprinellus*	11/21/2005	514028	4314645	15 N	670	16.3	-24.5
Bigmouth buffalo	*Ictiobus cyprinellus*	11/28/2005	510497	4310837	15 N	630	15.1	-24.7
Bigmouth buffalo	*Ictiobus cyprinellus*	11/28/2005	510497	4310837	15 N	595	15.6	-26.4
Bigmouth buffalo	*Ictiobus cyprinellus*	11/30/2005	515091	4314608	15 N	568	15.7	-25.6
Bigmouth buffalo	*Ictiobus cyprinellus*	12/22/2005	512633	4315014	15 N	611	15.9	-24.4
Bigmouth buffalo	*Ictiobus cyprinellus*	7/13/2006	508312	4310099	15 N	800	16.3	-25.7

Bigmouth buffalo	*Ictiobus cyprinellus*	7/13/2006	508312	4310099	15 N	554	15.9	-24.6
Bigmouth buffalo	*Ictiobus cyprinellus*	7/13/2006	508312	4310099	15 N	608	15.9	-26.7
Bigmouth buffalo	*Ictiobus cyprinellus*	7/13/2006	508312	4310099	15 N	578	17.2	-25.7
Bigmouth buffalo	*Ictiobus cyprinellus*	7/13/2006	508312	4310099	15 N	575	15.8	-25.0
Bigmouth buffalo	*Ictiobus cyprinellus*	7/13/2006	508312	4310099	15 N	545	16.3	-27.1
Bigmouth buffalo	*Ictiobus cyprinellus*	7/13/2006	508312	4310099	15 N	567	16.2	-25.5
Bigmouth buffalo	*Ictiobus cyprinellus*	7/13/2006	508312	4310099	15 N	460	16.8	-25.7
Bigmouth buffalo	*Ictiobus cyprinellus*	7/13/2006	508312	4310099	15 N	526	16.5	-25.7
Bighead carp	*Hypophthalmichthys nobilis*	11/21/2005	514028	4314645	15 N	702	18.4	-29.1
Bighead carp	*Hypophthalmichthys nobilis*	11/21/2005	514028	4314645	15 N	998	16.9	-23.4
Bighead carp	*Hypophthalmichthys nobilis*	11/21/2005	514028	4314645	15 N	701	17.4	-26.8
Bighead carp	*Hypophthalmichthys nobilis*	11/21/2005	514028	4314645	15 N	739	16.9	-26.5
Bighead carp	*Hypophthalmichthys nobilis*	11/21/2005	514028	4314645	15 N	695	16.9	-25.4
Bighead carp	*Hypophthalmichthys nobilis*	11/21/2005	514028	4314645	15 N	650	14.8	-29.0
Bighead carp	*Hypophthalmichthys nobilis*	11/21/2005	514028	4314645	15 N	665	16.9	-29.9
Bighead carp	*Hypophthalmichthys nobilis*	11/21/2005	514028	4314645	15 N	692	17.9	-28.9
Bighead carp	*Hypophthalmichthys nobilis*	11/21/2005	514028	4314645	15 N	715	19.4	-28.7
Bighead carp	*Hypophthalmichthys nobilis*	11/21/2005	514028	4314645	15 N	750	16.8	-25.3
Bighead carp	*Hypophthalmichthys nobilis*	11/21/2005	514028	4314645	15 N	710	16.7	-26.0
Bighead carp	*Hypophthalmichthys nobilis*	11/21/2005	514028	4314645	15 N	714	17.3	-27.0
Bighead carp	*Hypophthalmichthys nobilis*	11/21/2005	514028	4314645	15 N	704	17.2	-27.8
Bighead carp	*Hypophthalmichthys nobilis*	11/21/2005	514028	4314645	15 N	757	16.8	-26.5
Bighead carp	*Hypophthalmichthys nobilis*	11/21/2005	514028	4314645	15 N	697	17.7	-26.0
Bighead carp	*Hypophthalmichthys nobilis*	11/21/2005	514028	4314645	15 N	711	17.5	-27.4
Bighead carp	*Hypophthalmichthys nobilis*	11/21/2005	514028	4314645	15 N	663	18.0	-27.3
Bighead carp	*Hypophthalmichthys nobilis*	11/21/2005	514028	4314645	15 N	705	17.7	-27.5
Bighead carp	*Hypophthalmichthys nobilis*	11/21/2005	514028	4314645	15 N	732	17.0	-26.6
Bighead carp	*Hypophthalmichthys nobilis*	11/21/2005	514028	4314645	15 N	691	17.6	-26.5
Bighead carp	*Hypophthalmichthys nobilis*	11/21/2005	514028	4314645	15 N	709	16.4	-26.7
Bighead carp	*Hypophthalmichthys nobilis*	11/21/2005	514028	4314645	15 N	708	16.6	-27.1
Bighead carp	*Hypophthalmichthys nobilis*	11/21/2005	514028	4314645	15 N	736	15.7	-25.8
Bighead carp	*Hypophthalmichthys nobilis*	11/21/2005	514028	4314645	15 N	442	16.6	-25.7
Bighead carp	*Hypophthalmichthys nobilis*	11/21/2005	514028	4314645	15 N	770	17.9	-27.2
Bighead carp	*Hypophthalmichthys nobilis*	11/21/2005	514028	4314645	15 N	836	16.0	-26.3

Bighead carp	*Hypophthalmichthys nobilis*	11/21/2005	514028	4314645	15 N	734	17.1	-26.6
Bighead carp	*Hypophthalmichthys nobilis*	11/21/2005	514028	4314645	15 N	785	16.7	-25.8
Bighead carp	*Hypophthalmichthys nobilis*	11/21/2005	514028	4314645	15 N	705	18.1	-27.9
Bighead carp	*Hypophthalmichthys nobilis*	12/27/2005	589138	4269767	15 N	710	16.8	-26.4
Bighead carp	*Hypophthalmichthys nobilis*	12/27/2005	589138	4269767	15 N	1040	16.7	-29.2
Gizzard Shad	*Dorosoma cepedianum*	11/28/2005	510497	4310837	15 N	39	14.9	-25.0
Gizzard Shad	*Dorosoma cepedianum*	11/28/2005	510497	4310837	15 N	35	13.9	-25.3
Gizzard Shad	*Dorosoma cepedianum*	11/28/2005	510497	4310837	15 N	42	14.9	-24.3
Gizzard Shad	*Dorosoma cepedianum*	11/28/2005	510497	4310837	15 N	32	12.5	-32.5
Gizzard Shad	*Dorosoma cepedianum*	11/28/2005	510497	4310837	15 N	33	12.4	-29.4
Gizzard Shad	*Dorosoma cepedianum*	11/28/2005	510497	4310837	15 N	30.5	13.4	-22.9
Gizzard Shad	*Dorosoma cepedianum*	11/30/2005	515061	4314282	15 N	38	15.6	-24.6
Gizzard Shad	*Dorosoma cepedianum*	11/30/2005	515061	4314282	15 N	38	15.2	-23.6
Gizzard Shad	*Dorosoma cepedianum*	11/30/2005	515061	4314282	15 N	36	12.9	-31.4
Gizzard Shad	*Dorosoma cepedianum*	11/30/2005	515061	4314282	15 N	33.5	13.9	-24.0
Gizzard Shad	*Dorosoma cepedianum*	11/30/2005	515061	4314282	15 N	35	13.7	-25.6
Gizzard Shad	*Dorosoma cepedianum*	11/30/2005	515061	4314282	15 N	34	13.7	-25.5
Gizzard Shad	*Dorosoma cepedianum*	11/30/2005	515061	4314282	15 N	30.5	13.7	-24.7
Gizzard Shad	*Dorosoma cepedianum*	11/30/2005	515061	4314282	15 N	38	14.7	-23.7
Gizzard Shad	*Dorosoma cepedianum*	11/30/2005	515061	4314282	15 N	34	14.1	-23.2
Gizzard Shad	*Dorosoma cepedianum*	11/30/2005	515061	4314282	15 N	32	15.7	-31.8
Gizzard Shad	*Dorosoma cepedianum*	11/30/2005	515061	4314282	15 N	32.5	13.3	-29.5
Gizzard Shad	*Dorosoma cepedianum*	11/30/2005	515061	4314282	15 N	38.5	14.0	-31.1
Gizzard Shad	*Dorosoma cepedianum*	11/30/2005	515061	4314282	15 N	34	14.4	-26.2
Gizzard Shad	*Dorosoma cepedianum*	11/30/2005	515061	4314282	15 N	39.5	15.5	-27.8
Paddlefish	*Polyodon spathula*	11/21/2005	510490	4310838	15 N	746	15.6	-26.3
Paddlefish	*Polyodon spathula*	11/21/2005	514028	4314645	15 N	990	19.2	-29.4
Paddlefish	*Polyodon spathula*	11/21/2005	514028	4314645	15 N	865	18.3	-27.8
Paddlefish	*Polyodon spathula*	11/21/2005	514028	4314645	15 N	835	17.1	-30.9
Paddlefish	*Polyodon spathula*	11/28/2005	510497	4310837	15 N	615	15.6	-27.3
Paddlefish	*Polyodon spathula*	11/30/2005	515061	4314282	15 N	875	16.6	-28.5
Paddlefish	*Polyodon spathula*	12/27/2005	593201	4273587	15 N	960	19.6	-29.1
Paddlefish	*Polyodon spathula*	12/27/2005	593201	4273587	15 N	875	17.8	-28.5
Paddlefish	*Polyodon spathula*	12/27/2005	593507	4274020	15 N	785	14.9	-27.4

Paddlefish	*Polyodon spathula*	12/27/2005	593507	4274020	15 N	550	17.5	-28.2
Paddlefish	*Polyodon spathula*	12/27/2005	593507	4274020	15 N	820	18.0	-24.9
Paddlefish	*Polyodon spathula*	12/27/2005	593507	4274020	15 N	795	18.0	-28.6
Paddlefish	*Polyodon spathula*	12/27/2005	593507	4274020	15 N	680	16.7	-30.4
Paddlefish	*Polyodon spathula*	12/27/2005	593507	4274020	15 N	800	19.1	-27.3
Paddlefish	*Polyodon spathula*	12/27/2005	593507	4274020	15 N	780	15.2	-25.9
Pink Papershell	*Potamilus ohiensis*	11/27/2006	545316	4303133	15 N	48	11.5	-25.6
Pink Papershell	*Potamilus ohiensis*	11/27/2006	545316	4303133	15 N	36	11.2	-24.5
Pink Papershell	*Potamilus ohiensis*	11/28/2006	556150	4286479	15 N	74	12.4	-23.8
Pink Papershell	*Potamilus ohiensis*	11/28/2006	556150	4286479	15 N	66	12.2	-24.0
Pink Papershell	*Potamilus ohiensis*	11/28/2006	556150	4286479	15 N	59	12.0	-24.6
Pink Papershell	*Potamilus ohiensis*	11/28/2006	556150	4286479	15 N	58	12.0	-24.5
Pink Papershell	*Potamilus ohiensis*	11/28/2006	556150	4286479	15 N	53	12.0	-23.8
Pink Papershell	*Potamilus ohiensis*	11/28/2006	556150	4286479	15 N	52	10.9	-25.5
Pink Papershell	*Potamilus ohiensis*	11/28/2006	556150	4286479	15 N	43	11.2	-25.1
Pink Papershell	*Potamilus ohiensis*	11/28/2006	556150	4286479	15 N	45	11.3	-24.6
Pink Papershell	*Potamilus ohiensis*	11/28/2006	556150	4286479	15 N	45	12.4	-22.4
Pink Papershell	*Potamilus ohiensis*	11/28/2006	556150	4286479	15 N	51	11.2	-25.1

Data Directory:

Species = Species sampled, common name (Text)

Scientific name = Scientific name of species (Text)

Collection date = Date of collection (Date)

Easting = UTM coordinates for x (Numerical)

Northing = UTM coordinates for y (Numerical)

UTM Zone = UTM Zone (Text)

Length = total length of sample in mm, except for paddlefish, which are eye to fork length (Numerical)

$\delta^{15}N$ = ratio of nitrogen isotopes, (per mil) (Numerical)

$\delta^{13}C$= ratio of carbon isotopes, (per mil) (Numerical)
